# Does clopidogrel change morbidity and mortality in ICU sepsis patients?

**DOI:** 10.1186/cc13428

**Published:** 2014-03-17

**Authors:** DG Klepser, PP Dobesh, TR McGuire, DA Roberts, AL Himmelberg, KM Olsen

**Affiliations:** 1 University of Nebraska Medical Center College of Pharmacy, Omaha, NE, USA

## Introduction

The purpose of this study was to evaluate whether patients with sepsis exposed to clopidogrel have lower mortality and fewer days on mechanical ventilation compared with patients not exposed to clopidogrel. A recent post-hoc analysis of the PLATO trial suggests that the antiplatelet agent ticagrelor provided a significant reduction in sepsis-related mortality and pulmonary adverse events compared with clopidogrel. It is unknown whether clopidogrel also provides benefit in patients with sepsis compared with no therapy. Knowing this information may help focus future research on determining the mechanism of ticagrelor's benefit.

## Methods

This retrospective cohort study included all patients over the age of 40 with a confirmed diagnosis of severe sepsis and an ICU stay at our academic medical center from 1 January 2005 to 31 March 2011. Clopidogrel use, patient demographics, and APACHE II score at the time of sepsis were collected from patient charts. Clinical outcomes included in hospital mortality, development of acute respiratory distress syndrome (ARDS), days on mechanical ventilation, in-hospital cardiac events, hospital cost, and length of stay.

## Results

We identified 824 patients who met the inclusion criteria for this study. Of these patients, 76 (9.2%) had been exposed to clopidogrel. The mean APACHE II score was similar for patients receiving clopidogrel and those who did not (23.4 vs. 22.6; *P *= 0.426). Patients exposed to clopidogrel had a similar rate of in-hospital mortality (26.3% vs. 33.2%; *P *= 0.223) and ARDS (43.4% vs. 38.6%; *P *= 0.415). While mortality was also similar between the groups for patients with a low APACHE II score <25), mortality was lower in clopidogrel-exposed patients with an APACHE II score ≥25 (27.3% vs. 45.6%; *P *= 0.045). Patients exposed to clopidogrel did not have a higher use of blood products (65.8% vs. 65.9%; *P *= 0.983). Patients exposed to clopidogrel did not have significantly more days on mechanical ventilation, or ventilation-free days. Cardiac events during the hospital stay were not lower in patients on clopidogrel (4.0 vs. 2.4; *P *= 0.432). Hospital costs and length of stay did not differ between the groups.

## Conclusion

While clopidogrel may not increase the risk of bleeding in these patients, these data suggest that clopidogrel does not reduce adverse outcomes in patients with sepsis. A potential benefit in patients with APACHE II scores ≥25 may need further study. Future research into the mechanism of ticagrelor's benefit reported in the PLATO trial may be directed at the non-P2Y12 receptor mechanism.

